# P17 Retrospective review of inpatient antipseudomonal treatment using ciprofloxacin, piperacillin-tazobactam and meropenem in accordance with EUCAST criteria within an acute London Trust

**DOI:** 10.1093/jacamr/dlaf118.024

**Published:** 2025-07-14

**Authors:** Youngheun Kim, Shay Khan

**Affiliations:** Microbiology/Pharmacy Departments, Whittington Health NHS Trust, London, UK; Microbiology/Pharmacy Departments, Whittington Health NHS Trust, London, UK

## Abstract

**Background:**

*Pseudomonas aeruginosa (PsA)* is a common organism responsible for Healthcare-associated infections (HCAIs). The 23/24 English surveillance programme for antimicrobial utilization and resistance (ESPAUR) report showed *PsA* accounted for 7.2% of 1309 HCAIs. *PsA* is one of the most common Gram-negative MDR organisms found in hospitalized patients, contributing to prolonged hospital stays with a crude case fatality rate of 21.9%. Inappropriate dosing of antipseudomonal antibiotics can contribute to increased mortality, resistance, and treatment failure. EUCAST provides guidelines on ‘increased exposure dosing’ for effective treatment. This audit evaluates adherence to EUCAST dosing recommendations for ciprofloxacin (CIP), piperacillin-tazobactam (TZP), and meropenem (MER).

**Standards (target):**

Standard 1 (95%): Adherence to EUCAST criteria for CIP, TZP, and MER dosing in pseudomonal infections. Standard 2 (95%): Rate of appropriate dosing as per local guidance/microbiology advice. Standard 3 (95%): Rate of positive clinical outcomes (course completion with documentation of clinical improvement in clinical notes with no need for antimicrobial escalation). Standard 4 (95%): Rate of microbiology input.

**Secondary objectives:**

Analyse prescribing trends across specialties. Review local AMS practice and AMR rates.

**Methods:**

A retrospective audit was conducted over 3-months (01/08/24-31/10/24). Trust electronic prescribing and pathology systems were used to identify inpatients prescribed CIP, TZP and MER in response to pseudomonas isolates. Data analysis was undertaken by an antimicrobial pharmacist and compliance with four pre-defined standards was assessed.

**Results:**

In total, 43 inpatients were prescribed 64 antipseudomonals. TZP was the most commonly used antipseudomonal (35), followed by CIP (24) and MER (5). TZP: scored the lowest adherence to standard 1 (62.9%) but achieved 78.1% for standard 2 and 77.1% for standard 3. MER: had the highest adherence across all standards, 100% for 1 and 4, 80% for 2 and 3. CIP: 83.3% (standard 1), 70.1% (standard 2), 58.3% (standard 3). Respiratory was the highest users (39.1%), followed by elderly care (23.4%) and ITU (20.9%). TZP prescribing was poorest amongst respiratory (63.6% adherence to standards), compared to 100% adherence for CIP. ITU achieved 100% adherence for MER, CIP, TZP across all standards. The most commonly treated indications were pneumonia (21), urinary tract (15), skin and soft tissue (8) and bloodstream (6). Highest rate of resistance was found in sputum isolates: CIP (7%), TZP (18%) and MER (14%).

**Conclusions:**

The audit identified suboptimal adherence to EUCAST dosing recommendations, particularly for TZP. While microbiology input was consistently sought for restricted antimicrobials, overall compliance with correct dosing and duration standards fell below targets. This underscores the need for better prescriber education on ‘increased exposure dosing’ to improve patient outcomes and AMS. Limitations include sample size, short audit duration, and limited follow-up. Future efforts will focus on targeted multidisciplinary engagement and a re-audit to assess improvements.

